# A Complete Guide to Identify and Manage Malnutrition in Hospitalized Patients

**DOI:** 10.7759/cureus.8486

**Published:** 2020-06-07

**Authors:** Sohaip Kabashneh, Samer Alkassis, Layla Shanah, Hammad Ali

**Affiliations:** 1 Internal Medicine, Wayne State University/Detroit Medical Center, Detroit, USA

**Keywords:** malnutrition, prevalence, screening, diagnosis, management, oral nutritional supplements

## Abstract

Malnutrition is extremely common in hospitalized patients. It can lead to various complications and increase mortality. However, it remains poorly recognized and many health care systems do not require nutritional assessment during the hospital stay. This most likely due to lack of awareness and inadequate coordination between health care workers. Physicians can utilize many different methods when performing malnutrition screening, and there is also a lack of global clear-cut recommendations on criteria used to diagnose malnutrition. This article aims to increase malnutrition awareness among health care providers, and provide a guide on screening, diagnosis, and management of malnutrition.

## Introduction and background

There is no absolute agreement among societies on the definition of malnutrition, but frequently used elements in defining malnutrition are deficiencies of energy, protein, and a decrease in fat-free mass [1]. Malnutrition is a very common medical problem, affecting about half of the patients admitted to an acute hospital setting [2-4]. It increases the risk of negative health outcomes and complications including nosocomial infections, immunodeficiency, and pressure ulcers among others [5-7]. Despite its prevalence and negative impact on health outcomes, malnutrition remains poorly diagnosed and documented. Hence, dietary interventions and supplemental nutrition are underutilized thus compromising patient clinical outcomes [8].

The purpose of this review is to highlight the current literature available on the prevalence, impact on health outcomes, screening, and diagnosis of malnutrition, in addition to providing recommendations on the management of malnutrition and the benefit of nutritional supplements in malnourished patients.

## Review

Prevalence of malnutrition on admission and the risk of nosocomial malnutrition

Malnutrition is a common problem; it includes both undernutrition (underweight) and overnutrition (overweight and obesity). This public health epidemic targets all spectrums of the population; however, the most vulnerable groups are those with low-socioeconomic status, older people, those with acute or chronic illnesses, and expectant mothers [9].

Lim et al. conducted a study to estimate the prevalence of malnutrition on admission in a tertiary hospital in Singapore involving 818 adults. Malnutrition was evident in 29% [2]. To evaluate the prevalence of malnutrition in England, Edington et al. evaluated 850 patients, and malnutrition on admission was found in 20% [3]. In the Netherlands, Naber et al. found that 45% of patients hospitalized for internal or gastrointestinal diseases were malnourished on admission [4]. Hence, the prevalence of malnutrition varies depending on the geographic location and population being studied. Studies show malnutrition is found in approximately 20 to 50 percent of hospitalized adults.

Malnutrition is often present upon hospital admission. Additionally, 38% of well-nourished inpatients and 69% of malnourished inpatients either develop malnutrition or suffer further deterioration of nutritional status during hospitalization [10-12]. Nosocomial malnutrition is a potentially preventable cause of poor outcomes. Unfortunately, it often goes unrecognized by healthcare providers.

Malnutrition is associated with a significant increase in morbidity and mortality in the hospital setting

Malnutrition was found to increase total complications in a study of 709 adults. The incidence of complications in the malnourished group was 27% compared to only 16.8% in the well-nourished group [5]. The same study found that malnutrition also increases the length of hospital stay; malnourished patients had a median length of stay of nine days compared to only six days in the well‐nourished patients [5]. Similarly, results from a study of 173 hospitalized patients found that the median length of stay for patients at risk for malnutrition was six days compared to four days for well‐nourished patients [13].

In addition to a prolonged hospital stay, a study of 837 patients over a 14-month period found that 25% of malnourished subjects required readmission to a healthcare facility after being discharged compared with 11% of the well-nourished group [14]. Correia and Waitzberg showed that hospital mortality in the malnourished patients was higher (12.4%) compared to 4.7% in the well-nourished, with a relative risk of 2.63 [5]. In addition to hospital mortality, studies showed that protein-energy undernutrition is a strong risk factor for mortality during the subsequent 4.5 years [15].

Malnutrition is also associated with altered immune responses, as many studies showed that malnourished individuals are at a higher risk of developing nosocomial infections. They had a higher incidence of sepsis and intra‐abdominal abscess [5,6,16]. Malnourished patients also have a higher risk of developing pneumonia and have a higher risk of mortality from pneumonia [17].

The occurrence of pressure ulcers is also higher in patients with malnutrition. Studies showed that compared to well‐nourished patients, malnourished patients are 2.1 times more likely to develop decubitus ulcers [7].

Malnutrition can exaggerate age‐related reduced muscle mass, which subsequently leads to deconditioning [18]. In fact, studies have shown that most elderly inpatients with hospital-associated deconditioning are malnourished. Likewise, malnutrition is associated with poor rehabilitation outcome in hospital-associated deconditioning [19].

Malnutrition was also associated with higher hospital costs. Braunschweig et al. conducted a study on 404 adults and found that patients who declined nutritionally, regardless of nutritional status at admission, had significantly higher hospital charges ($45,762) compared to those who did not ($28,631) [10]. Similarly, Correia and Waitzberg showed that malnutrition was associated with up to a threefold increase in hospital costs [5].

Inadequate malnutrition identification and diagnosis

Failure to identify and subsequently refer to a dietitian lead to the persistently high prevalence of malnutrition. Several studies have been conducted to investigate malnutrition recognition and documentation in the hospital setting in an attempt to combat malnutrition.

Among the studies conducted, Gout et al. reported that only 15% of malnourished patients were correctly identified and documented, and a dietitian was involved in only 45% of malnutrition cases [8]. Kellett et al. reported that the prevalence of malnutrition is found to be 52%. Unfortunately, only 5.4% of patients were coded as malnourished which is most likely due to lack of identification [10].

Nutrition screening for hospitalized patients

In January 2016, the Global Leadership Initiative on Malnutrition (GLIM) reached a consensus to use a two‐step approach for the diagnosis of malnutrition - first screening to identify “at-risk” status, and second assessment for diagnosis and grading the severity of malnutrition [20].

An important step in combating malnutrition is to increase screening in order to identify patients that are malnourished and/or at high risk of developing malnutrition during their hospital course. Usability is key in choosing a screening tool. The Nutrition Risk Screening 2002 (NRS-2002) and Mini Nutritional Assessment (MNA) rely on a few questions, do not require professional nutrition expertise, and do not take a long time to complete, thus, are the preferred screening tools in the healthcare setting and are recommended by the European Society for Clinical Nutrition and Metabolism (ESPEN). The following is an overview of both the tools in a healthcare setting [21].

The Hospital: Nutrition Risk Screening 2002

NRS-2002 is the preferred tool to detect undernutrition and the risk of developing undernutrition in the hospital setting [21,22]. It includes a pre-screening questionnaire (Table 1). If the patient answers ‘Yes’ to any of the pre-screening questions, then the actual screening (Table 2) is indicated; otherwise, the patient is re-screened at weekly intervals [23].

**Table 1:** NRS-2002 pre-screening questions

NRS-2002 pre-screening questions
Is BMI <20.5?
Has the patient lost weight within the last 3 months?
Has the patient had a reduced dietary intake in the last week?
Is the patient severely ill? (e.g., in intensive therapy)

NRS-2002 actual screening uses two main categories impairment of nutritional status and increases in requirements to identify patients at nutritional risk (Table 2). If age-corrected total ≥3 then comprehensive nutritional evaluation and subsequent intervention are indicated; otherwise, the screening is repeated weekly [23].

**Table 2:** Nutritional risk screening (NRS 2002) ^a^ Body Mass Index ^b^ Chronic Obstructive Pulmonary Disease

Nutritional risk screening (NRS 2002)			
Impaired nutritional status		Severity of disease	
Score 0	Normal nutritional status	Score 0	Normal nutritional requirements
Score 1	Weight loss >5% in 3 months or food intake below 50–75% of normal requirement in the preceding week	Score 1	Hip fracture, chronic patients particularly with acute complications: cirrhosis, COPD^b^, chronic hemodialysis, diabetes, oncology
Score 2	Weight loss >5% in 2 months or BMI^a^ 18.5–20.5 + impaired general condition or Food intake 25–50% of normal requirement in preceding week	Score 2	Major abdominal surgery, stroke, severe pneumonia, hematologic malignancy
Score 3	Weight loss >5% in 1 month (>15% in 3 months) or BMI <18.5 + impaired general condition or food intake 0–25% of normal requirement in the preceding week.	Score 3	Head injury, bone marrow transplantation, intensive care patients (APACHE 10)
To calculate the total score: 1. Find score (0–3) for impaired nutritional status and severity of disease. 2. Add the two scores (→ total score) 3. If age ≥70 years: add 1 to the total score to correct for the frailty of elderly.			

The Elderly in Nursing Homes and Hospitals: Mini Nutritional Assessment

**Figure 1:**
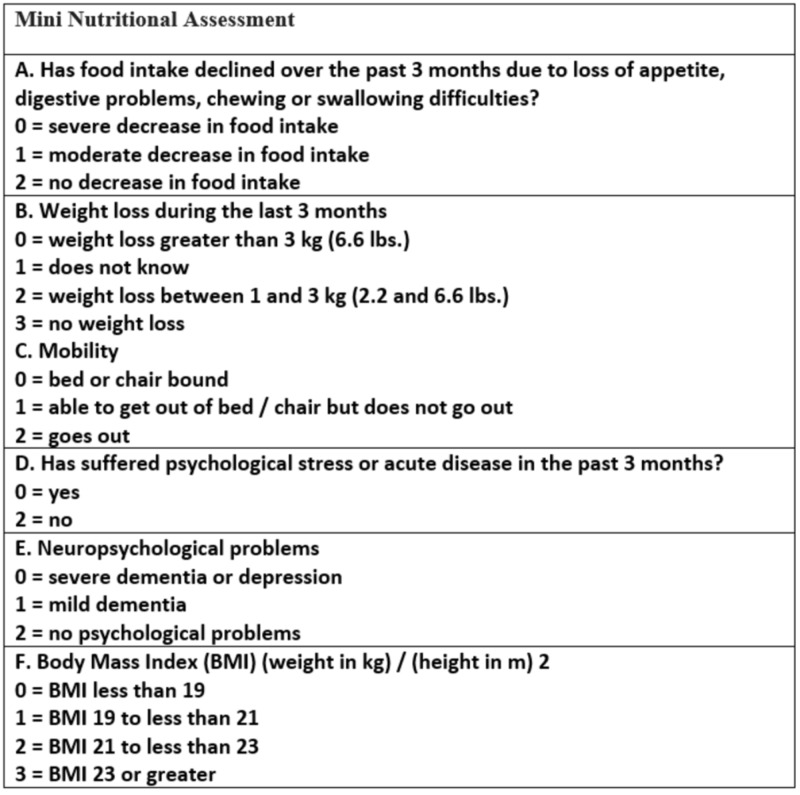
Mini nutritional assessment

Mini nutritional assessment (MNA) is used to detect undernutrition and the risk of developing undernutrition among the older population living in nursing homes and hospitals [21]. The MNA is more likely to identify the risk of developing undernutrition at an early stage in the frail elderly because it looks at the physical and mental aspects that affect the nutritional status of the elderly (Figure 1). The patient is evaluated with six questions, a score of eleven points or below warrants further assessment and appropriate nutritional plan [24].

Malnutrition diagnosis

GLIM reached a consensus on the criteria to be used when making the diagnosis of malnutrition. It includes three phenotypic criteria (weight loss, low body mass index, and reduced muscle mass ), and two etiologic criteria (reduced food intake or assimilation and disease burden). For the diagnosis of malnutrition, a combination of at least one phenotypic criterion and one etiologic criterion is required (Table 3). Because it is clinically useful to identify the severity of malnutrition, GLIM also developed phenotypic metrics for grading severity as moderate and severe (Table 4) [20].

**Table 3:** Phenotypic and etiologic criteria for the diagnosis of malnutrition. GI = gastro-intestinal, ER = energy requirements. Adapted with permission from [20]. Copyright © 2018 Elsevier Ltd, the European Society for Clinical Nutrition and Metabolism and American Society for Parenteral and Enteral Nutrition.

Phenotypic Criteria			Etiologic Criteria	
Weight loss (%)	Low body mass index (kg/m^2^)	Reduced muscle mass	Reduced food intake or assimilation	Inflammation
>5% within past 6 months, or >10% beyond 6 months	<20 if <70 years, or <22 if >70 years	Reduced by validated body composition measuring techniques	≤50% of ER > 1 week, or any reduction for >2 weeks, or any chronic GI condition that adversely impacts food assimilation or absorption	Acute disease/injury or chronic disease-related
	Asia: <18.5 if <70 years, or <20 if >70 years			

**Table 4:** Grading the severity of malnutrition. Adapted with permission from [20]. Copyright © 2018 Elsevier Ltd, the European Society for Clinical Nutrition and Metabolism and American Society for Parenteral and Enteral Nutrition.

Phenotypic Criteria			
	Weight loss (%)	Low body mass index (kg/m^2^)	Reduced muscle mass
Stage 1/Moderate Malnutrition (Requires 1 phenotypic criterion that meets this grade)	5-10% within the past 6 months, or 10-20% beyond 6 months	<20 if <70 years, <22 if ≥70 years	Mild to moderate deficit (per validated assessment methods)
Stage 2/Severe Malnutrition (Requires 1 phenotypic criterion that meets this grade)	>10% within the past 6 months, or >20% beyond 6 months	<18.5 if <70 years, <20 if ≥70 years	Severe deficit (per validated assessment methods)

Management of malnutrition

A diagnosis of malnutrition should be followed by a consultation with skilled nutrition practitioners like dietitians for comprehensive nutritional assessments if possible. After a complete assessment is performed, nutritional requirements can be calculated and a plan to meet those requirements is initiated.

Oral nutritional supplements (ONS) can be used if improvements in energy, protein, and micronutrient intakes are required. An overview of 13 systematic reviews and meta-analyses by Stratton and Elia found that ONS were associated with significant clinical benefits. In the study, the daily intake of ONS was between 250 and 600 kcal/day, the duration of supplementation varied from a short period in hospital (one week) to a prolonged period in the community (up to two years) [25].

The reviews by Stratton and Elia suggest that ONS consistently improved total nutritional intake, with little suppression of food intake [25]. Thus, it has a positive effect on body weight, significantly attenuating weight loss in the acutely ill. It also showed a significant reduction in mortality particularly in acutely ill elderly [25]. High protein ONS was associated with a lower risk of pressure ulcers in high-risk groups (frail elderly, hip fracture, poor mobility) [26]. A three-month intervention with ONS appears to be cost-effective according to international benchmarks [27].

## Conclusions

Malnutrition is an exceedingly common medical problem with significant effects on morbidity and mortality. Despite its significance, it is underdiagnosed in healthcare systems. In this review, we recommend nutrition screening by either NRS-2002 for hospitalized patients or MNA for the older population living in nursing homes. Positive screening should be followed by GLIM criteria evaluation for diagnosis and severity grading. Consultation of skilled nutrition practitioners is needed when the diagnosis is made for full nutritional evaluation and calorie count. ONS has a positive effect on body weight and decreases mortality and should be considered in the management of malnutrition.
